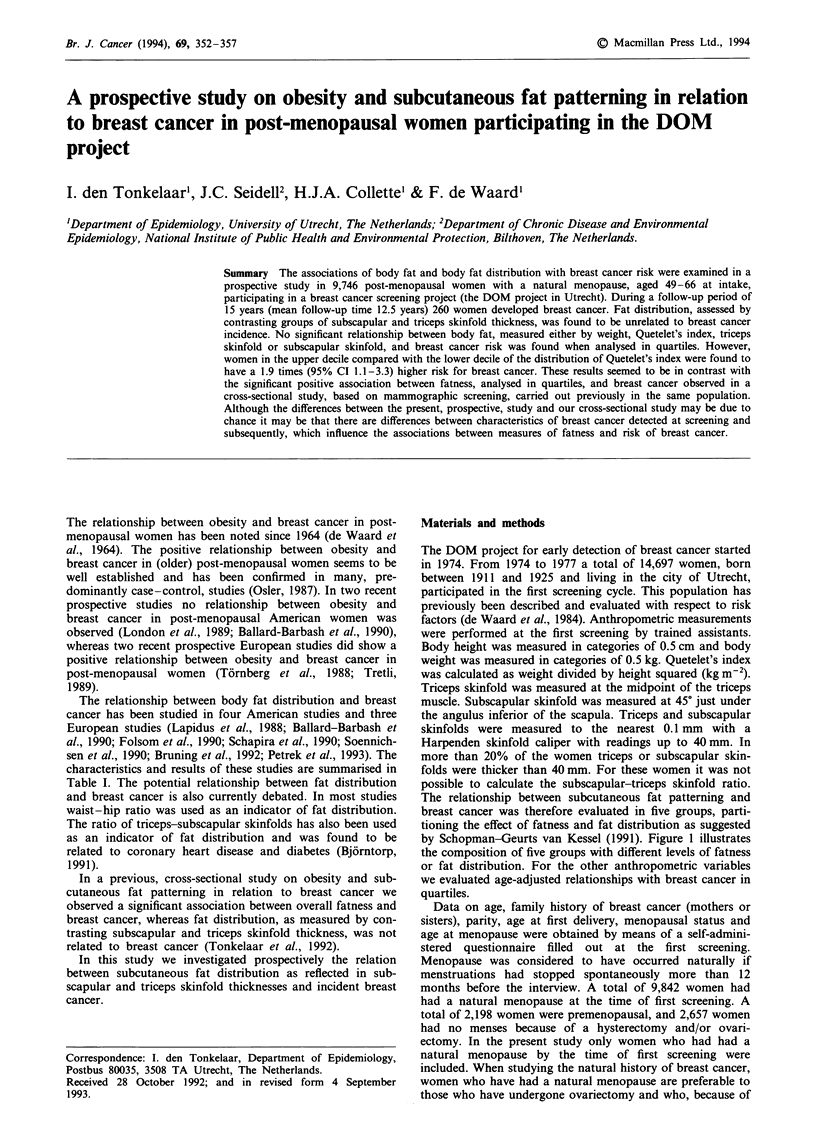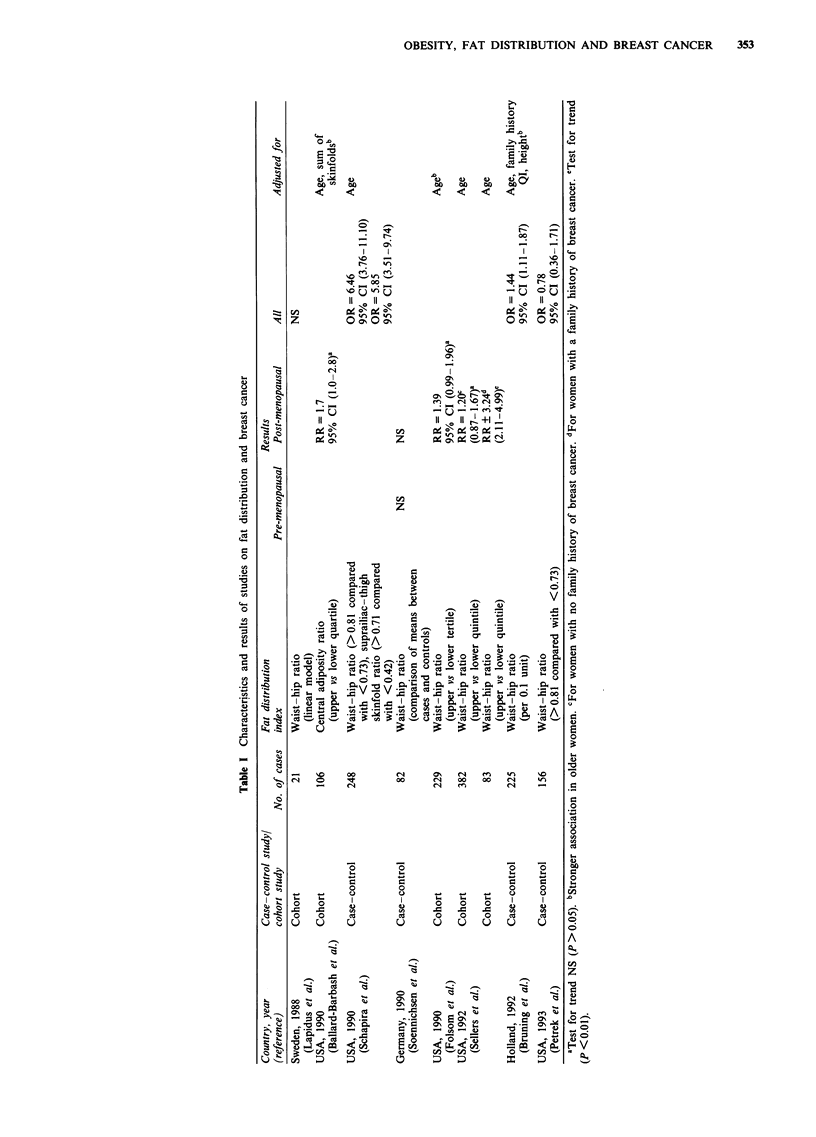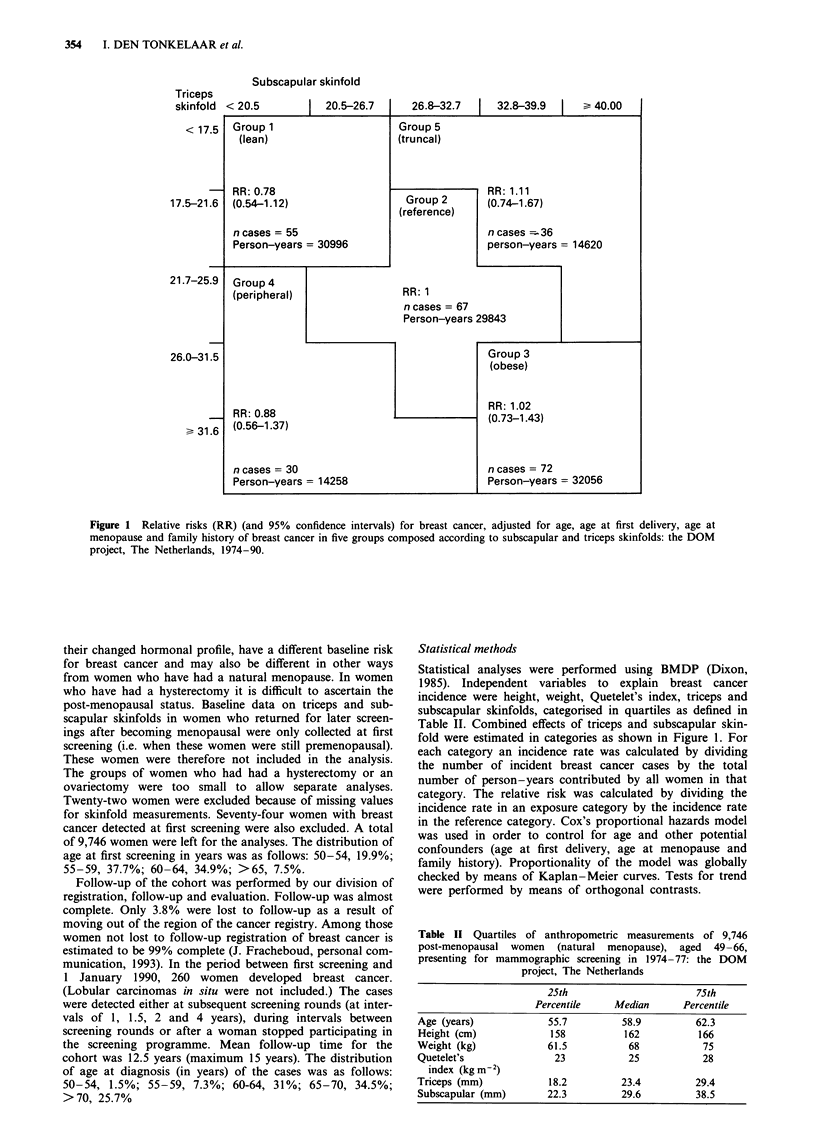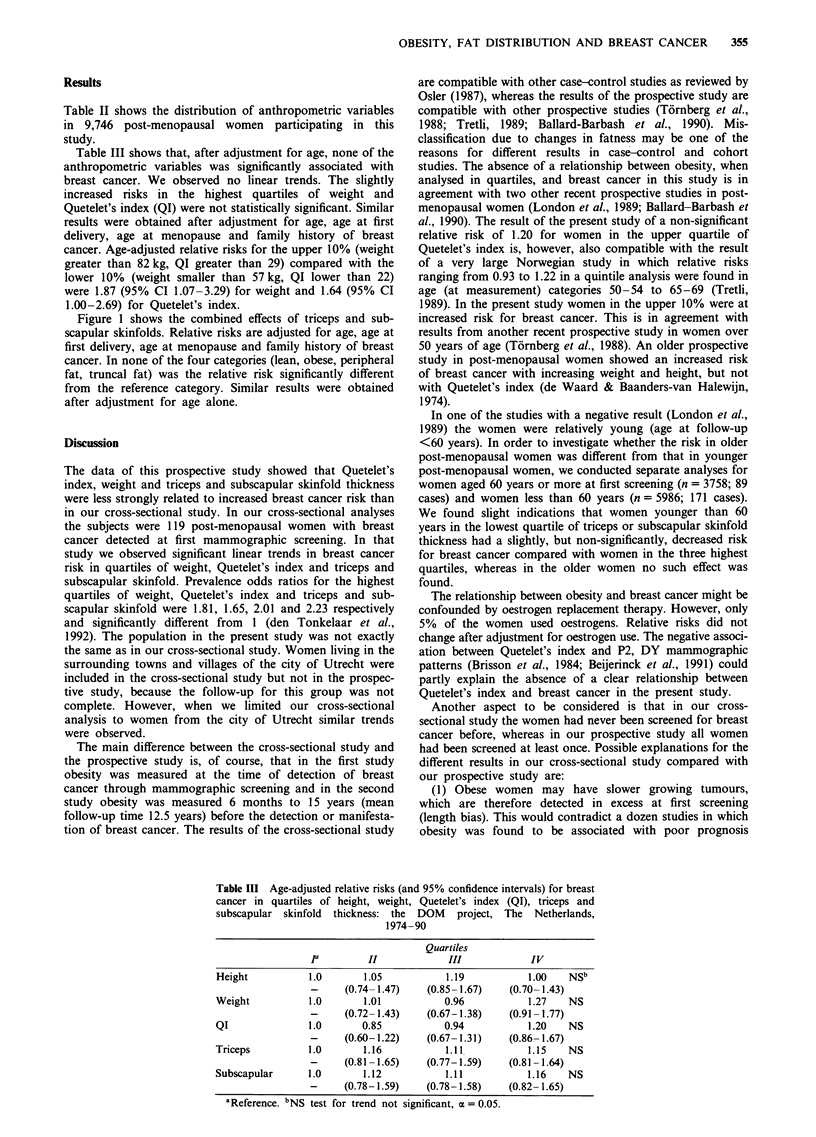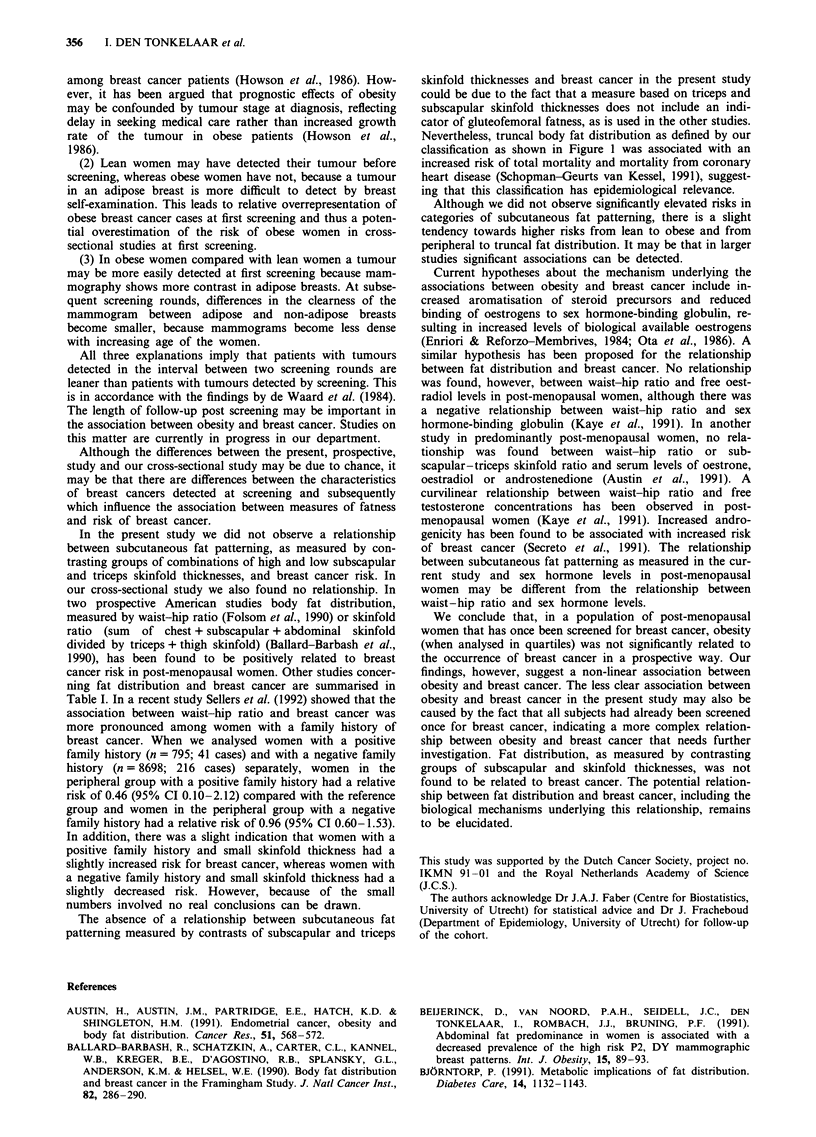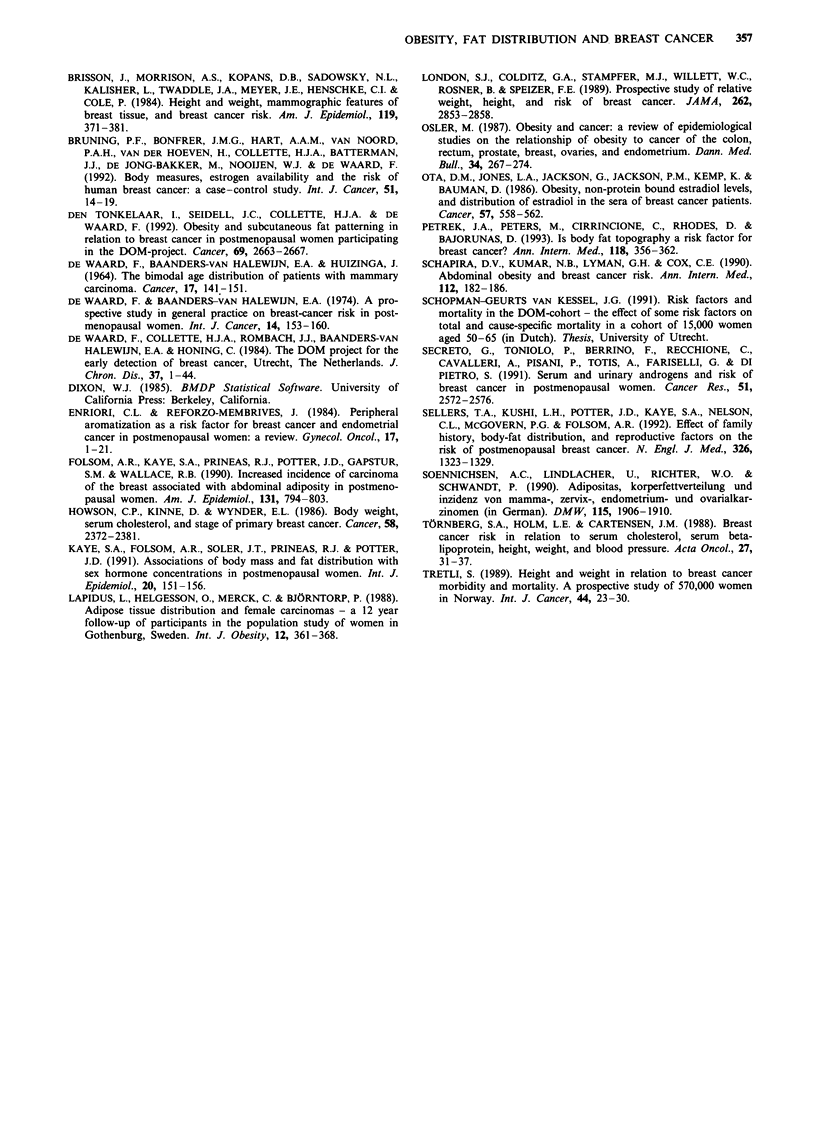# A prospective study on obesity and subcutaneous fat patterning in relation to breast cancer in post-menopausal women participating in the DOM project.

**DOI:** 10.1038/bjc.1994.64

**Published:** 1994-02

**Authors:** I. den Tonkelaar, J. C. Seidell, H. J. Collette, F. de Waard

**Affiliations:** Department of Epidemiology, University of Utrecht, The Netherlands.

## Abstract

The associations of body fat and body fat distribution with breast cancer risk were examined in a prospective study in 9,746 post-menopausal women with a natural menopause, aged 49-66 at intake, participating in a breast cancer screening project (the DOM project in Utrecht). During a follow-up period of 15 years (mean follow-up time 12.5 years) 260 women developed breast cancer. Fat distribution, assessed by contrasting groups of subcapsular and triceps skinfold thickness, was found to be unrelated to breast cancer incidence. No significant relationship between body fat, measured either by weight, Quetelet's index, triceps skinfold or subscapular skinfold, and breast cancer risk was found when analysed in quartiles. However, women in the upper decile compared with the lower decile of the distribution of Quetelet's index were found to have a 1.9 times (95% CI 1.1-3.3) higher risk for breast cancer. These results seemed to be in contrast with the significant positive association between fatness, analysed in quartiles, and breast cancer observed in a cross-sectional study, based on mammographic screening, carried out previously in the same population. Although the differences between the present, prospective, study and our cross-sectional study may be due to chance it may be that there are differences between characteristics of breast cancer detected at screening and subsequently, which influence the associations between measures of fatness and risk of breast cancer.


Br. J. Cancer (1994), 69, 352-357 © Macmillan Press Ltd., 1994

### A prospective study on obesity and subcutaneous fat patterning in relation to breast cancer in post-menopausal women participating in the DOM project

I. den Tonkelaar[1], J.C. Seidell[2], H.J.A. Collette[1] & F. de Waard[1]

[1]*Department of Epidemiology, University of Utrecht, The Netherlands;* [2]*Department of Chronic Disease and Environmental Epidemiology, National Institute of Public Health and Environmental Protection, Bilthoven, The Netherlands.*

**Summary** The associations of body fat and body fat distribution with breast cancer risk were examined in a prospective study in 9,746 post-menopausal women with a natural menopause, aged 49-66 at intake, participating in a breast cancer screening project (the DOM project in Utrecht). During a follow-up period of 15 years (mean follow-up time 12.5 years) 260 women developed breast cancer. Fat distribution, assessed by contrasting groups of subscapular and triceps skinfold thickness, was found to be unrelated to breast cancer incidence. No significant relationship between body fat, measured either by weight, Quetelet's index, triceps skinfold or subscapular skinfold, and breast cancer risk was found when analysed in quartiles. However, women in the upper decile compared with the lower decile of the distribution of Quetelet's index were found to have a 1.9 times (95% CI 1.1-3.3) higher risk for breast cancer. These results seemed to be in contrast with the significant positive association between fatness, analysed in quartiles, and breast cancer observed in a cross-sectional study, based on mammographic screening, carried out previously in the same population. Although the differences between the present, prospective, study and our cross-sectional study may be due to chance it may be that there are differences between characteristics of breast cancer detected at screening and subsequently, which influence the associations between measures of fatness and risk of breast cancer.

The relationship between obesity and breast cancer in post-menopausal women has been noted since 1964 (de Waard *et al.*, 1964). The positive relationship between obesity and breast cancer in (older) post-menopausal women seems to be well established and has been confirmed in many, predominantly case-control, studies (Osler, 1987). In two recent prospective studies no relationship between obesity and breast cancer in post-menopausal American women was observed (London *et al.*, 1989; Ballard-Barbash *et al.*, 1990), whereas two recent prospective European studies did show a positive relationship between obesity and breast cancer in post-menopausal women (Törnberg *et al.*, 1988; Tretli, 1989).

The relationship between body fat distribution and breast cancer has been studied in four American studies and three European studies (Lapidus *et al.*, 1988; Ballard-Barbash *et al.*, 1990; Folsom *et al.*, 1990; Schapira *et al.*, 1990; Soennichsen *et al.*, 1990; Bruning *et al.*, 1992; Petrek *et al.*, 1993). The characteristics and results of these studies are summarised in Table I. The potential relationship between fat distribution and breast cancer is also currently debated. In most studies waist-hip ratio was used as an indicator of fat distribution. The ratio of triceps-subscapular skinfolds has also been used as an indicator of fat distribution and was found to be related to coronary heart disease and diabetes (Björntorp, 1991).

In a previous, cross-sectional study on obesity and subcutaneous fat patterning in relation to breast cancer we observed a significant association between overall fatness and breast cancer, whereas fat distribution, as measured by contrasting subscapular and triceps skinfold thickness, was not related to breast cancer (Tonkelaar *et al.*, 1992).

In this study we investigated prospectively the relation between subcutaneous fat distribution as reflected in subscapular and triceps skinfold thicknesses and incident breast cancer.

Correspondence: I. den Tonkelaar, Department of Epidemiology, Postbus 80035, 3508 TA Utrecht, The Netherlands.
Received 28 October 1992; and in revised form 4 September 1993.

### Materials and methods

The DOM project for early detection of breast cancer started in 1974. From 1974 to 1977 a total of 14,697 women, born between 1911 and 1925 and living in the city of Utrecht, participated in the first screening cycle. This population has previously been described and evaluated with respect to risk factors (de Waard *et al.*, 1984). Anthropometric measurements were performed at the first screening by trained assistants. Body height was measured in categories of 0.5 cm and body weight was measured in categories of 0.5 kg. Quetelet's index was calculated as weight divided by height squared (kg $m^{-2}$). Triceps skinfold was measured at the midpoint of the triceps muscle. Subscapular skinfold was measured at 45° just under the angulus inferior of the scapula. Triceps and subscapular skinfolds were measured to the nearest 0.1 mm with a Harpenden skinfold caliper with readings up to 40 mm. In more than 20% of the women triceps or subscapular skinfolds were thicker than 40 mm. For these women it was not possible to calculate the subscapular-triceps skinfold ratio. The relationship between subcutaneous fat patterning and breast cancer was therefore evaluated in five groups, partitioning the effect of fatness and fat distribution as suggested by Schopman-Geurts van Kessel (1991). Figure 1 illustrates the composition of five groups with different levels of fatness or fat distribution. For the other anthropometric variables we evaluated age-adjusted relationships with breast cancer in quartiles.

Data on age, family history of breast cancer (mothers or sisters), parity, age at first delivery, menopausal status and age at menopause were obtained by means of a self-administered questionnaire filled out at the first screening. Menopause was considered to have occurred naturally if menstruations had stopped spontaneously more than 12 months before the interview. A total of 9,842 women had had a natural menopause at the time of first screening. A total of 2,198 women were premenopausal, and 2,657 women had no menses because of a hysterectomy and/or ovariectomy. In the present study only women who had had a natural menopause by the time of first screening were included. When studying the natural history of breast cancer, women who have had a natural menopause are preferable to those who have undergone ovariectomy and who, because of

**Table I** Characteristics and results of studies on fat distribution and breast cancer

| *Country, year (reference)* | *Case–control study/ cohort study* | *No. of cases* | *Fat distribution index* | *Results* Pre-menopausal | Post-menopausal | *All* | *Adjusted for* |
|---|---|---|---|---|---|---|---|
| Sweden, 1988 (Lapidus *et al.*) | Cohort | 21 | Waist–hip ratio (linear model) | | | NS | |
| USA, 1990 (Ballard-Barbash *et al.*) | Cohort | 106 | Central adiposity ratio (upper *vs* lower quartile) | | RR = 1.7 95% CI (1.0–2.8)[a] | | Age, sum of skinfolds[b] |
| USA, 1990 (Schapira *et al.*) | Case–control | 248 | Waist–hip ratio ($>0.81$ compared with $<0.73$), suprailiac–thigh skinfold ratio ($>0.71$ compared with $<0.42$) | | | OR = 6.46 95% CI (3.76–11.10) OR = 5.85 95% CI (3.51–9.74) | Age |
| Germany, 1990 (Soennichsen *et al.*) | Case–control | 82 | Waist–hip ratio (comparison of means between cases and controls) | NS | NS | | |
| USA, 1990 (Folsom *et al.*) | Cohort | 229 | Waist–hip ratio (upper *vs* lower tertile) | | RR = 1.39 95% CI (0.99–1.96)[a] | | Age[b] |
| USA, 1992 (Sellers *et al.*) | Cohort | 382 | Waist–hip ratio (upper *vs* lower quintile) | | RR = 1.20[c] (0.87–1.67)[a] | | Age |
| | Cohort | 83 | Waist–hip ratio (upper *vs* lower quintile) | | RR ± 3.24[d] (2.11–4.99)[e] | | Age |
| Holland, 1992 (Bruning *et al.*) | Case–control | 225 | Waist–hip ratio (per 0.1 unit) | | | OR = 1.44 95% CI (1.11–1.87) | Age, family history QI, height[b] |
| USA, 1993 (Petrek *et al.*) | Case–control | 156 | Waist–hip ratio ($>0.81$ compared with $<0.73$) | | | OR = 0.78 95% CI (0.36–1.71) | |

[a]Test for trend NS ($P > 0.05$). [b]Stronger association in older women. [c]For women with no family history of breast cancer. [d]For women with a family history of breast cancer. [e]Test for trend ($P < 0.01$).

**Figure 1** Relative risks (RR) (and 95% confidence intervals) for breast cancer, adjusted for age, age at first delivery, age at menopause and family history of breast cancer in five groups composed according to subscapular and triceps skinfolds: the DOM project, The Netherlands, 1974–90.

their changed hormonal profile, have a different baseline risk for breast cancer and may also be different in other ways from women who have had a natural menopause. In women who have had a hysterectomy it is difficult to ascertain the post-menopausal status. Baseline data on triceps and subscapular skinfolds in women who returned for later screenings after becoming menopausal were only collected at first screening (i.e. when these women were still premenopausal). These women were therefore not included in the analysis. The groups of women who had had a hysterectomy or an ovariectomy were too small to allow separate analyses. Twenty-two women were excluded because of missing values for skinfold measurements. Seventy-four women with breast cancer detected at first screening were also excluded. A total of 9,746 women were left for the analyses. The distribution of age at first screening in years was as follows: 50–54, 19.9%; 55–59, 37.7%; 60–64, 34.9%; >65, 7.5%.

Follow-up of the cohort was performed by our division of registration, follow-up and evaluation. Follow-up was almost complete. Only 3.8% were lost to follow-up as a result of moving out of the region of the cancer registry. Among those women not lost to follow-up registration of breast cancer is estimated to be 99% complete (J. Fracheboud, personal communication, 1993). In the period between first screening and 1 January 1990, 260 women developed breast cancer. (Lobular carcinomas *in situ* were not included.) The cases were detected either at subsequent screening rounds (at intervals of 1, 1.5, 2 and 4 years), during intervals between screening rounds or after a woman stopped participating in the screening programme. Mean follow-up time for the cohort was 12.5 years (maximum 15 years). The distribution of age at diagnosis (in years) of the cases was as follows: 50–54, 1.5%; 55–59, 7.3%; 60-64, 31%; 65–70, 34.5%; >70, 25.7%

### *Statistical methods*

Statistical analyses were performed using BMDP (Dixon, 1985). Independent variables to explain breast cancer incidence were height, weight, Quetelet's index, triceps and subscapular skinfolds, categorised in quartiles as defined in Table II. Combined effects of triceps and subscapular skinfold were estimated in categories as shown in Figure 1. For each category an incidence rate was calculated by dividing the number of incident breast cancer cases by the total number of person–years contributed by all women in that category. The relative risk was calculated by dividing the incidence rate in an exposure category by the incidence rate in the reference category. Cox's proportional hazards model was used in order to control for age and other potential confounders (age at first delivery, age at menopause and family history). Proportionality of the model was globally checked by means of Kaplan–Meier curves. Tests for trend were performed by means of orthogonal contrasts.

**Table II** Quartiles of anthropometric measurements of 9,746 post-menopausal women (natural menopause), aged 49–66, presenting for mammographic screening in 1974–77: the DOM project, The Netherlands

| | *25th Percentile* | *Median* | *75th Percentile* |
|---|---|---|---|
| Age (years) | 55.7 | 58.9 | 62.3 |
| Height (cm) | 158 | 162 | 166 |
| Weight (kg) | 61.5 | 68 | 75 |
| Quetelet's index (kg $m^{-2}$) | 23 | 25 | 28 |
| Triceps (mm) | 18.2 | 23.4 | 29.4 |
| Subscapular (mm) | 22.3 | 29.6 | 38.5 |

### Results

Table II shows the distribution of anthropometric variables in 9,746 post-menopausal women participating in this study.

Table III shows that, after adjustment for age, none of the anthropometric variables was significantly associated with breast cancer. We observed no linear trends. The slightly increased risks in the highest quartiles of weight and Quetelet's index (QI) were not statistically significant. Similar results were obtained after adjustment for age, age at first delivery, age at menopause and family history of breast cancer. Age-adjusted relative risks for the upper 10% (weight greater than 82 kg, QI greater than 29) compared with the lower 10% (weight smaller than 57 kg, QI lower than 22) were 1.87 (95% CI 1.07–3.29) for weight and 1.64 (95% CI 1.00–2.69) for Quetelet's index.

Figure 1 shows the combined effects of triceps and subscapular skinfolds. Relative risks are adjusted for age, age at first delivery, age at menopause and family history of breast cancer. In none of the four categories (lean, obese, peripheral fat, truncal fat) was the relative risk significantly different from the reference category. Similar results were obtained after adjustment for age alone.

### Discussion

The data of this prospective study showed that Quetelet's index, weight and triceps and subscapular skinfold thickness were less strongly related to increased breast cancer risk than in our cross-sectional study. In our cross-sectional analyses the subjects were 119 post-menopausal women with breast cancer detected at first mammographic screening. In that study we observed significant linear trends in breast cancer risk in quartiles of weight, Quetelet's index and triceps and subscapular skinfold. Prevalence odds ratios for the highest quartiles of weight, Quetelet's index and triceps and subscapular skinfold were 1.81, 1.65, 2.01 and 2.23 respectively and significantly different from 1 (den Tonkelaar *et al.*, 1992). The population in the present study was not exactly the same as in our cross-sectional study. Women living in the surrounding towns and villages of the city of Utrecht were included in the cross-sectional study but not in the prospective study, because the follow-up for this group was not complete. However, when we limited our cross-sectional analysis to women from the city of Utrecht similar trends were observed.

The main difference between the cross-sectional study and the prospective study is, of course, that in the first study obesity was measured at the time of detection of breast cancer through mammographic screening and in the second study obesity was measured 6 months to 15 years (mean follow-up time 12.5 years) before the detection or manifestation of breast cancer. The results of the cross-sectional study are compatible with other case–control studies as reviewed by Osler (1987), whereas the results of the prospective study are compatible with other prospective studies (Törnberg *et al.*, 1988; Tretli, 1989; Ballard-Barbash *et al.*, 1990). Misclassification due to changes in fatness may be one of the reasons for different results in case–control and cohort studies. The absence of a relationship between obesity, when analysed in quartiles, and breast cancer in this study is in agreement with two other recent prospective studies in post-menopausal women (London *et al.*, 1989; Ballard–Barbash *et al.*, 1990). The result of the present study of a non-significant relative risk of 1.20 for women in the upper quartile of Quetelet's index is, however, also compatible with the result of a very large Norwegian study in which relative risks ranging from 0.93 to 1.22 in a quintile analysis were found in age (at measurement) categories 50–54 to 65–69 (Tretli, 1989). In the present study women in the upper 10% were at increased risk for breast cancer. This is in agreement with results from another recent prospective study in women over 50 years of age (Törnberg *et al.*, 1988). An older prospective study in post-menopausal women showed an increased risk of breast cancer with increasing weight and height, but not with Quetelet's index (de Waard & Baanders-van Halewijn, 1974).

In one of the studies with a negative result (London *et al.*, 1989) the women were relatively young (age at follow-up <60 years). In order to investigate whether the risk in older post-menopausal women was different from that in younger post-menopausal women, we conducted separate analyses for women aged 60 years or more at first screening ($n = 3758$; 89 cases) and women less than 60 years ($n = 5986$; 171 cases). We found slight indications that women younger than 60 years in the lowest quartile of triceps or subscapular skinfold thickness had a slightly, but non-significantly, decreased risk for breast cancer compared with women in the three highest quartiles, whereas in the older women no such effect was found.

The relationship between obesity and breast cancer might be confounded by oestrogen replacement therapy. However, only 5% of the women used oestrogens. Relative risks did not change after adjustment for oestrogen use. The negative association between Quetelet's index and P2, DY mammographic patterns (Brisson *et al.*, 1984; Beijerinck *et al.*, 1991) could partly explain the absence of a clear relationship between Quetelet's index and breast cancer in the present study.

Another aspect to be considered is that in our cross-sectional study the women had never been screened for breast cancer before, whereas in our prospective study all women had been screened at least once. Possible explanations for the different results in our cross-sectional study compared with our prospective study are:

(1) Obese women may have slower growing tumours, which are therefore detected in excess at first screening (length bias). This would contradict a dozen studies in which obesity was found to be associated with poor prognosis

**Table III** Age-adjusted relative risks (and 95% confidence intervals) for breast cancer in quartiles of height, weight, Quetelet's index (QI), triceps and subscapular skinfold thickness: the DOM project, The Netherlands, 1974–90

| | Quartiles *I*[a] | *II* | *III* | *IV* | |
|---|---|---|---|---|---|
| Height | 1.0 | 1.05 | 1.19 | 1.00 | NS[b] |
| | – | (0.74–1.47) | (0.85–1.67) | (0.70–1.43) | |
| Weight | 1.0 | 1.01 | 0.96 | 1.27 | NS |
| | – | (0.72–1.43) | (0.67–1.38) | (0.91–1.77) | |
| QI | 1.0 | 0.85 | 0.94 | 1.20 | NS |
| | – | (0.60–1.22) | (0.67–1.31) | (0.86–1.67) | |
| Triceps | 1.0 | 1.16 | 1.11 | 1.15 | NS |
| | – | (0.81–1.65) | (0.77–1.59) | (0.81–1.64) | |
| Subscapular | 1.0 | 1.12 | 1.11 | 1.16 | NS |
| | – | (0.78–1.59) | (0.78–1.58) | (0.82–1.65) | |

[a]Reference. [b]NS test for trend not significant, $\alpha = 0.05$.

among breast cancer patients (Howson *et al.*, 1986). However, it has been argued that prognostic effects of obesity may be confounded by tumour stage at diagnosis, reflecting delay in seeking medical care rather than increased growth rate of the tumour in obese patients (Howson *et al.*, 1986).

(2) Lean women may have detected their tumour before screening, whereas obese women have not, because a tumour in an adipose breast is more difficult to detect by breast self-examination. This leads to relative overrepresentation of obese breast cancer cases at first screening and thus a potential overestimation of the risk of obese women in cross-sectional studies at first screening.

(3) In obese women compared with lean women a tumour may be more easily detected at first screening because mammography shows more contrast in adipose breasts. At subsequent screening rounds, differences in the clearness of the mammogram between adipose and non-adipose breasts become smaller, because mammograms become less dense with increasing age of the women.

All three explanations imply that patients with tumours detected in the interval between two screening rounds are leaner than patients with tumours detected by screening. This is in accordance with the findings by de Waard *et al.* (1984). The length of follow-up post screening may be important in the association between obesity and breast cancer. Studies on this matter are currently in progress in our department.

Although the differences between the present, prospective, study and our cross-sectional study may be due to chance, it may be that there are differences between the characteristics of breast cancers detected at screening and subsequently which influence the association between measures of fatness and risk of breast cancer.

In the present study we did not observe a relationship between subcutaneous fat patterning, as measured by contrasting groups of combinations of high and low subscapular and triceps skinfold thicknesses, and breast cancer risk. In our cross-sectional study we also found no relationship. In two prospective American studies body fat distribution, measured by waist–hip ratio (Folsom *et al.*, 1990) or skinfold ratio (sum of chest + subscapular + abdominal skinfold divided by triceps + thigh skinfold) (Ballard–Barbash *et al.*, 1990), has been found to be positively related to breast cancer risk in post-menopausal women. Other studies concerning fat distribution and breast cancer are summarised in Table I. In a recent study Sellers *et al.* (1992) showed that the association between waist–hip ratio and breast cancer was more pronounced among women with a family history of breast cancer. When we analysed women with a positive family history ($n = 795$; 41 cases) and with a negative family history ($n = 8698$; 216 cases) separately, women in the peripheral group with a positive family history had a relative risk of 0.46 (95% CI 0.10–2.12) compared with the reference group and women in the peripheral group with a negative family history had a relative risk of 0.96 (95% CI 0.60–1.53). In addition, there was a slight indication that women with a positive family history and small skinfold thickness had a slightly increased risk for breast cancer, whereas women with a negative family history and small skinfold thickness had a slightly decreased risk. However, because of the small numbers involved no real conclusions can be drawn.

The absence of a relationship between subcutaneous fat patterning measured by contrasts of subscapular and triceps skinfold thicknesses and breast cancer in the present study could be due to the fact that a measure based on triceps and subscapular skinfold thicknesses does not include an indicator of gluteofemoral fatness, as is used in the other studies. Nevertheless, truncal body fat distribution as defined by our classification as shown in Figure 1 was associated with an increased risk of total mortality and mortality from coronary heart disease (Schopman–Geurts van Kessel, 1991), suggesting that this classification has epidemiological relevance.

Although we did not observe significantly elevated risks in categories of subcutaneous fat patterning, there is a slight tendency towards higher risks from lean to obese and from peripheral to truncal fat distribution. It may be that in larger studies significant associations can be detected.

Current hypotheses about the mechanism underlying the associations between obesity and breast cancer include increased aromatisation of steroid precursors and reduced binding of oestrogens to sex hormone-binding globulin, resulting in increased levels of biological available oestrogens (Enriori & Reforzo–Membrives, 1984; Ota *et al.*, 1986). A similar hypothesis has been proposed for the relationship between fat distribution and breast cancer. No relationship was found, however, between waist–hip ratio and free oestradiol levels in post-menopausal women, although there was a negative relationship between waist–hip ratio and sex hormone-binding globulin (Kaye *et al.*, 1991). In another study in predominantly post-menopausal women, no relationship was found between waist–hip ratio or subscapular–triceps skinfold ratio and serum levels of oestrone, oestradiol or androstenedione (Austin *et al.*, 1991). A curvilinear relationship between waist–hip ratio and free testosterone concentrations has been observed in post-menopausal women (Kaye *et al.*, 1991). Increased androgenicity has been found to be associated with increased risk of breast cancer (Secreto *et al.*, 1991). The relationship between subcutaneous fat patterning as measured in the current study and sex hormone levels in post-menopausal women may be different from the relationship between waist–hip ratio and sex hormone levels.

We conclude that, in a population of post-menopausal women that has once been screened for breast cancer, obesity (when analysed in quartiles) was not significantly related to the occurrence of breast cancer in a prospective way. Our findings, however, suggest a non-linear association between obesity and breast cancer. The less clear association between obesity and breast cancer in the present study may also be caused by the fact that all subjects had already been screened once for breast cancer, indicating a more complex relationship between obesity and breast cancer that needs further investigation. Fat distribution, as measured by contrasting groups of subscapular and skinfold thicknesses, was not found to be related to breast cancer. The potential relationship between fat distribution and breast cancer, including the biological mechanisms underlying this relationship, remains to be elucidated.

This study was supported by the Dutch Cancer Society, project no. IKMN 91-01 and the Royal Netherlands Academy of Science (J.C.S.).

The authors acknowledge Dr J.A.J. Faber (Centre for Biostatistics, University of Utrecht) for statistical advice and Dr J. Fracheboud (Department of Epidemiology, University of Utrecht) for follow-up of the cohort.

### References

AUSTIN, H., AUSTIN, J.M., PARTRIDGE, E.E., HATCH, K.D. & SHINGLETON, H.M. (1991). Endometrial cancer, obesity and body fat distribution. *Cancer Res.*, **51**, 568–572.

BALLARD-BARBASH, R., SCHATZKIN, A., CARTER, C.L., KANNEL, W.B., KREGER, B.E., D'AGOSTINO, R.B., SPLANSKY, G.L., ANDERSON, K.M. & HELSEL, W.E. (1990). Body fat distribution and breast cancer in the Framingham Study. *J. Natl Cancer Inst.*, **82**, 286–290.

BEIJERINCK, D., VAN NOORD, P.A.H., SEIDELL, J.C., DEN TONKELAAR, I., ROMBACH, J.J., BRUNING, P.F. (1991). Abdominal fat predominance in women is associated with a decreased prevalence of the high risk P2, DY mammographic breast patterns. *Int. J. Obesity*, **15**, 89–93.

BJÖRNTORP, P. (1991). Metabolic implications of fat distribution. *Diabetes Care*, **14**, 1132–1143.

BRISSON, J., MORRISON, A.S., KOPANS, D.B., SADOWSKY, N.L., KALISHER, L., TWADDLE, J.A., MEYER, J.E., HENSCHKE, C.I. & COLE, P. (1984). Height and weight, mammographic features of breast tissue, and breast cancer risk. *Am. J. Epidemiol.*, **119**, 371-381.

BRUNING, P.F., BONFRER, J.M.G., HART, A.A.M., VAN NOORD, P.A.H., VAN DER HOEVEN, H., COLLETTE, H.J.A., BATTERMAN, J.J., DE JONG-BAKKER, M., NOOIJEN, W.J. & DE WAARD, F. (1992). Body measures, estrogen availability and the risk of human breast cancer: a case-control study. *Int. J. Cancer*, **51**, 14-19.

DEN TONKELAAR, I., SEIDELL, J.C., COLLETTE, H.J.A. & DE WAARD, F. (1992). Obesity and subcutaneous fat patterning in relation to breast cancer in postmenopausal women participating in the DOM-project. *Cancer*, **69**, 2663-2667.

DE WAARD, F., BAANDERS-VAN HALEWIJN, E.A. & HUIZINGA, J. (1964). The bimodal age distribution of patients with mammary carcinoma. *Cancer*, **17**, 141-151.

DE WAARD, F. & BAANDERS-VAN HALEWIJN, E.A. (1974). A prospective study in general practice on breast-cancer risk in postmenopausal women. *Int. J. Cancer*, **14**, 153-160.

DE WAARD, F., COLLETTE, H.J.A., ROMBACH, J.J., BAANDERS-VAN HALEWIJN, E.A. & HONING, C. (1984). The DOM project for the early detection of breast cancer, Utrecht, The Netherlands. *J. Chron. Dis.*, **37**, 1-44.

DIXON, W.J. (1985). *BMDP Statistical Software*. University of California Press: Berkeley, California.

ENRIORI, C.L. & REFORZO-MEMBRIVES, J. (1984). Peripheral aromatization as a risk factor for breast cancer and endometrial cancer in postmenopausal women: a review. *Gynecol. Oncol.*, **17**, 1-21.

FOLSOM, A.R., KAYE, S.A., PRINEAS, R.J., POTTER, J.D., GAPSTUR, S.M. & WALLACE, R.B. (1990). Increased incidence of carcinoma of the breast associated with abdominal adiposity in postmenopausal women. *Am. J. Epidemiol.*, **131**, 794-803.

HOWSON, C.P., KINNE, D. & WYNDER, E.L. (1986). Body weight, serum cholesterol, and stage of primary breast cancer. *Cancer*, **58**, 2372-2381.

KAYE, S.A., FOLSOM, A.R., SOLER, J.T., PRINEAS, R.J. & POTTER, J.D. (1991). Associations of body mass and fat distribution with sex hormone concentrations in postmenopausal women. *Int. J. Epidemiol.*, **20**, 151-156.

LAPIDUS, L., HELGESSON, O., MERCK, C. & BJÖRNTORP, P. (1988). Adipose tissue distribution and female carcinomas - a 12 year follow-up of participants in the population study of women in Gothenburg, Sweden. *Int. J. Obesity*, **12**, 361-368.

LONDON, S.J., COLDITZ, G.A., STAMPFER, M.J., WILLETT, W.C., ROSNER, B. & SPEIZER, F.E. (1989). Prospective study of relative weight, height, and risk of breast cancer. *JAMA*, **262**, 2853-2858.

OSLER, M. (1987). Obesity and cancer: a review of epidemiological studies on the relationship of obesity to cancer of the colon, rectum, prostate, breast, ovaries, and endometrium. *Dann. Med. Bull.*, **34**, 267-274.

OTA, D.M., JONES, L.A., JACKSON, G., JACKSON, P.M., KEMP, K. & BAUMAN, D. (1986). Obesity, non-protein bound estradiol levels, and distribution of estradiol in the sera of breast cancer patients. *Cancer*, **57**, 558-562.

PETREK, J.A., PETERS, M., CIRRINCIONE, C., RHODES, D. & BAJORUNAS, D. (1993). Is body fat topography a risk factor for breast cancer? *Ann. Intern. Med.*, **118**, 356-362.

SCHAPIRA, D.V., KUMAR, N.B., LYMAN, G.H. & COX, C.E. (1990). Abdominal obesity and breast cancer risk. *Ann. Intern. Med.*, **112**, 182-186.

SCHOPMAN-GEURTS VAN KESSEL, J.G. (1991). Risk factors and mortality in the DOM-cohort - the effect of some risk factors on total and cause-specific mortality in a cohort of 15,000 women aged 50-65 (in Dutch). *Thesis*, University of Utrecht.

SECRETO, G., TONIOLO, P., BERRINO, F., RECCHIONE, C., CAVALLERI, A., PISANI, P., TOTIS, A., FARISELLI, G. & DI PIETRO, S. (1991). Serum and urinary androgens and risk of breast cancer in postmenopausal women. *Cancer Res.*, **51**, 2572-2576.

SELLERS, T.A., KUSHI, L.H., POTTER, J.D., KAYE, S.A., NELSON, C.L., MCGOVERN, P.G. & FOLSOM, A.R. (1992). Effect of family history, body-fat distribution, and reproductive factors on the risk of postmenopausal breast cancer. *N. Engl. J. Med.*, **326**, 1323-1329.

SOENNICHSEN, A.C., LINDLACHER, U., RICHTER, W.O. & SCHWANDT, P. (1990). Adipositas, korperfettverteilung und inzidenz von mamma-, zervix-, endometrium- und ovarialkarzinomen (in German). *DMW*, **115**, 1906-1910.

TÖRNBERG, S.A., HOLM, L.E. & CARTENSEN, J.M. (1988). Breast cancer risk in relation to serum cholesterol, serum betalipoprotein, height, weight, and blood pressure. *Acta Oncol.*, **27**, 31-37.

TRETLI, S. (1989). Height and weight in relation to breast cancer morbidity and mortality. A prospective study of 570,000 women in Norway. *Int. J. Cancer*, **44**, 23-30.